# Effect of Immersion in Water or Alkali Solution on the Structures and Properties of Epoxy Resin

**DOI:** 10.3390/polym13121902

**Published:** 2021-06-08

**Authors:** Bin Wang, Dihui Li, Guijun Xian, Chenggao Li

**Affiliations:** 1 Central Research Institute of Building and Construction Co., Ltd., Beijing 100088, China; mccwangbin@126.com; 2 Key Lab of Structures Dynamic Behavior and Control, Ministry of Education, Harbin Institute of Technology, Harbin 150090, China; lidihui1992@163.com (D.L.); gjxian@hit.edu.cn (G.X.); 3 Key Lab of Smart Prevention and Mitigation of Civil Engineering Disasters of the Ministry of Industry and Information Technology, Harbin Institute of Technology, Harbin 150090, China; 4 School of Civil Engineering, Harbin Institute of Technology, Harbin 150090, China

**Keywords:** epoxy resin, water or alkali solution, water uptake, structural evolution, tensile strength

## Abstract

The durability of fiber-reinforced polymer (FRP) composites is significantly dependent on the structures and properties of the resin matrix. In the present paper, the effects of physical or chemical interactions between the molecular chain of the epoxy resin matrix and water molecules or alkaline groups on the water absorption, mechanical structures, and microstructures of epoxy resin samples were studied experimentally. The results showed that the water uptake curves of the epoxy resin immersed in water and an alkali solution over time presented a three-stage variation. At different immersion stages, the water uptake behavior of the resin showed unique characteristics owing to the coupling effects of the solution concentration gradient diffusion, molecular hydrolysis reaction, and molecular segment movement. In comparison with the water immersion, the alkali solution environment promoted the hydrolysis reaction of the epoxy resin molecular chain. After the immersion in water or the alkali solution for one month, the water uptake of the resin was close to saturate, and the viscoelasticity was observed to decrease significantly. The micropore and free volume space on the surface and in the interior of the resin gradually increased, while the original large-scale free volume space decreased. The tensile strength decreased to the lowest point after the immersion in water and the alkali solution for one month, and the decrease percentages at 20 °C and 60 °C water or 60 °C alkali solution were 24%, 28%, and 22%, respectively. Afterward, the tensile strength recovered with the further extension of immersion time. In addition, it can be found that the effect of the alkali solution and water on the tensile strength of the epoxy resin was basically the same.

## 1. Introduction

Fiber-reinforced polymer (FRP) composite is a new type of structural material designed to replace steel products in the field of civil engineering. Owing to excellent characteristics, such as light weight, high strength, and corrosion resistance, FRP can be used alone or in combination with other structural materials such as steel and concrete. Facing complex civil service environments (such as salt, alkali, marine, and other corrosive environments), the durability evaluation of FRP exposed to these environments is of great significance to guarantee the safety and reliability of engineering application [1,2,3]. At present, the duration of FRP in engineering application is relatively short. The method for effectively evaluating the properties of FRP before engineering applications is still an important subject to be clarified [4,5,6,7,8].

Compared with the reinforcement fiber, the resin matrix (especially for epoxy resin) has multiformity in type and composition. Furthermore, the molecular chain structure is susceptible to thermosetting reaction conditions and other factors after the curing of the resin, which makes research on the durability of FRP more complicated and difficult. This study focused on the environmental aging resistance of the epoxy resin matrix, including long-term exposure to humidity, temperature, water/salt or alkali solution, etc. It was found that the property evolution of the resin matrix was mainly dependent on the water absorption, and the effect of water molecules on chemical reactions included the destruction of the original group (such as the crosslinking fracture of the molecular segment) and the generation of microcracks and micropores from the swelling of the resin matrix, which had complex effects on the macro mechanical properties of the resin [9,10,11]. Therefore, the evaluation of structures and properties evolution of epoxy resin exposed to the above service environments is significant to provide rapid laboratory testing and long-life evaluation of epoxy resin for engineering applications.

Some researchers have found that the water absorption of resin was mainly affected by the free volume [12,13]. Others proposed that the hydrophilic functional groups in the molecular chain of the resin had a significant effect on the water absorption [14,15,16]. The Fickian diffusion model is usually used to describe the process of water absorption. However, due to the chemical reaction, process conditions, and external environment, the water absorption behavior of epoxy becomes more complicated. Some studies revealed that water molecules entered into the resin and caused plasticization, and this plasticization can be basically recovered after drying. However, the degradation of macro mechanical properties caused by the molecular chain breakage and the microcracks caused by hydrolysis are irreversible [17,18]. The above mechanisms can be used for reference to study the durability of epoxy resin.

Relevant research on the durability of fiber-reinforced composites showed that the fiber/resin interface was susceptible to water molecules, which led to the degradation of interface bonding properties and the macro properties of composites. The interface bonding properties of the composites were influenced both by the chemical bonding structure and the surface physical structure of the fiber and resin. Especially in the process of water absorption, the effects of hygroscopic expansion and surface pores of the resin matrix on the interfacial bonding have not been deeply studied, and the mechanism of hygroscopic degradation of composites is not clear.

In the present study, the epoxy resin was selected and the curing process was effectively controlled. The cured epoxy resin was immersed in water and an alkali solution at 20 °C, 40 °C, and 60 °C for different times. The selection of alkali solution was used to simulate the actual pore solution of concrete (pH = 12.5). The variation of water uptake, chemical structure, thermal properties, strain, and free volume of epoxy resin with the immersion time, and the change and distribution of free volume before and after drying, were compared. The characteristics of chemical and physical structure changes of epoxy resin with the immersion time were analyzed from the perspectives of solution concentration gradient diffusion, hydrolysis reaction, plasticization, and the time-dependent relaxation process of molecular segment movement. The influence of structural evolution of epoxy resin on its tensile strength was revealed, which could provide reference for rapid detection and evaluation of epoxy resin in engineering applications, as well as for durability research and life evaluations of epoxy resin matrix composites.

## 2. Materials and Methods 

### 2.1. Raw Materials and Sample Preparation

**Figure 1:**
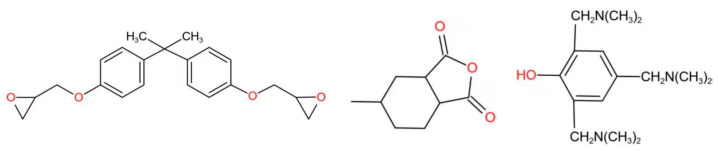
Molecular structures of components of epoxy resin.

The bisphenol A epoxy (DGEBA), with the abbreviation of E51 (Phoenix brand, Wuxi Chemical Plant), was selected as the resin matrix with a density of 1.2 g/cm^3^, the curing agent was methylhexahydrophthalic anhydride (MeHHPA), and the curing accelerator was 2, 4, 6-tris (dimethylaminomethyl) phenol (DMP30). The molecular structures of the above three components are shown in Figure 1, and the mass ratio was 100:80:2. The molecular weights of the monomers of DGEBA, MeHHPA, and DMP30 were 340, 168, and 265, respectively. 

The E51 epoxy was preheated to 60 °C for 30 min and then the curing agent and accelerator were added according to the above ratio. The mixed solution was then evenly stirred with a glass rod for 10 min at room temperature and subsequently oscillated with ultrasonic waves for 15 min to remove the bubbles from the stirring process. An aluminum mold with a cavity size of 200 mm × 150 mm × 4 mm was preheated to 120 °C. The epoxy resin was poured into the mold and cured at 120 °C for 90 min, then heated to 150 °C for 90 min, and finally cooled to room temperature. The tensile strength, tensile modulus, and elongation at break of epoxy were 62.08 MPa, 2.59 GPa, and 2.4%, respectively. 

After the curing, the resin blocks were cut into different sizes according to the experimental settings. The samples were polished with a polishing machine to remove surface chips and defects so as to ensure the uniform size.

### 2.2. Water Uptake Test

The epoxy samples were fully immersed in two kinds of solutions, a distilled water solution and an alkali solution (simulating a mature concrete pore solution with pH = 12.5). The samples were placed in a water bath and kept at constant temperatures. According to ACI 440.3R-04, the alkali solution was prepared with 118.5 g of Ca(OH)_2_, 0.9 g of NaOH, and 4.2 g of KOH in 1000 g of distilled water. The immersion temperatures were 20 °C, 40 °C, and 60 °C respectively, and the immersion times were 0 h, 4 h, 8 h, 24 h, 3 d, 1 w, 1 month (m), 2 m, 3 m, and 5 m. 

The sample size for the water absorption test was 25 mm × 25 mm × 2 mm. Before immersion, the samples were dried in a 60 °C oven for 2 days. The weights of the samples, swiped of water on the surface, were measured with an electronic balance with an accuracy of 0.1 mg. A total of 5 samples for each condition were weighed, and the average value was calculated. The water uptake of epoxy was obtained as follows:

(1) $$W(\%)=\frac{W_{t}-W_{0}}{W_{0}}\times 100\%$$

where *W_t_* is the wet weight at time *t*, and *W*_0_ is the initial weight before the immersion. 

### 2.3. FTIR Test

Fourier transform infrared spectroscopy (FTIR) (AVATAR 360, Nicolet, WI, USA) was used to measure IR absorption of the initial and immersed samples in the two solutions for 5 months. The water on the surface of the sample was dried and then the sample was grinded into powder with a mass of 2 mg by mixing KBr powder in a ratio of 1:100. The mixture was then pressed into a pallet.

### 2.4. DMA Test

A dynamic thermodynamic analysis (DMTA) test (Q800, TA, USA) was performed for the epoxy resin specimens with size of 40 mm × 8 mm × 2 mm using tensile film mode with 1 Hz frequency during heating from 20 °C to 200 °C at 5 °C/min.

### 2.5. Internal Strain Test

During the preparation process of the epoxy resin, in a silicone rubber mold with a size of 50 mm × 50 mm × 2 mm, two fiber gratings were embedded in the epoxy resin. One was directly in contact with the resin to monitor the wavelength variation of the grating from the immersed temperature and resin stress. The other one was used to monitor the wavelength variation of the grating from the immersed temperature and was encapsulated with a capillary steel tube to protect the grating. The temperature and strain data were obtained according to the acquisition of wavelength variation data by the fiber Bragg grating acquisition instrument. The relationship between temperature, strain, and grating wavelength can be expressed as follows [19]:

(2) $$\Delta \lambda_{1}=\alpha_{\varepsilon }\Delta \varepsilon +\alpha_{T1}\Delta T$$

(3) $$\Delta \lambda_{2}=\alpha_{T2}\Delta T$$

(4) $$\Delta \lambda =\Delta \lambda_{1}-K\Delta \lambda_{2}=\alpha_{\varepsilon }\Delta \varepsilon$$

where Δλ_1_ is the value of grating wavelength change, Δ*ε* is the value of strain change, Δ*T* is the value of temperature change, *α_T_*_1_ is the temperature sensitivity coefficient of grating 1 (its value is 10 pm/°C), *α_T_*_2_ is the temperature sensitivity coefficient of grating 2 (its value is 10 pm/°C), and *α*_ε_ is the strain sensitivity coefficient (1.2 pm/με). When the temperature has slight changes, α_𝑇_ has little change and *K* value could be taken as one. The fiber grating demodulator adopted KNPFBG produced by Shanghai Qipeng Engineering Materials Technology Co., Ltd. (Shanghai, China), with the resolution of 1 pm and data sampling frequency of 10 Hz.

### 2.6. Positron Annihilation Lifetime Spectra (PALS) Test

Positron annihilation lifetime spectra (PALS) tests of the initial and immersed samples for 5 months were conducted using a multichannel analyzer data buffer (Ortec Adcam mode) with a time resolution of 0.27 ns full width at half-maximum. The probe is made by a BaF_2_ crystal and an XP2020Q PMT. Approximately 20 mg of Ci Na^22^Cl was directly deposited onto the epoxy resin specimens, which were sandwiched between two identical samples with size of 15 mm × 15 mm × 1.5 mm. All PALS spectra were measured at room temperature. Every spectrum was obtained over a period of approximately 2 h and contained about 10^6^ integrated counts. 

Three positron lifetimes were resolved for the spectra. The longest lifetime component consisted of the lifetime of *τ*_3_ and the corresponding intensity of *I*_3_, which was usually attributed to the ortho-positronium (o-Ps) pickoff annihilation in the free volume holes. The lifetime *τ*_3_ and the intensity *I*_3_ were related to the size and concentration of free volume holes, respectively. The lifetime *τ*_3_ was defined as a function of the average radius R of free volume holes, as follows [20,21]:

(5) $$\tau_{3}=\frac{1}{2}{\left[1-\frac{R}{R+\Delta R}+\frac{1}{2\pi }\sin\left(\frac{2\pi R}{R+\Delta R}\right)\right]}^{-1}$$

where Δ*R* (equal to 0.1656) was obtained by fitting the observed o-Ps lifetime in molecular solids with a known hole size. The apparent free volume fraction is defined as [21,22]

(6) $$f_{app}=VI_{3}=\frac{4}{3}\pi R^{3}I_{3}$$

where *f_app_* is the free volume fraction and 𝑉 is the volume of free volume.

### 2.7. Tensile Test

**Figure 2:**
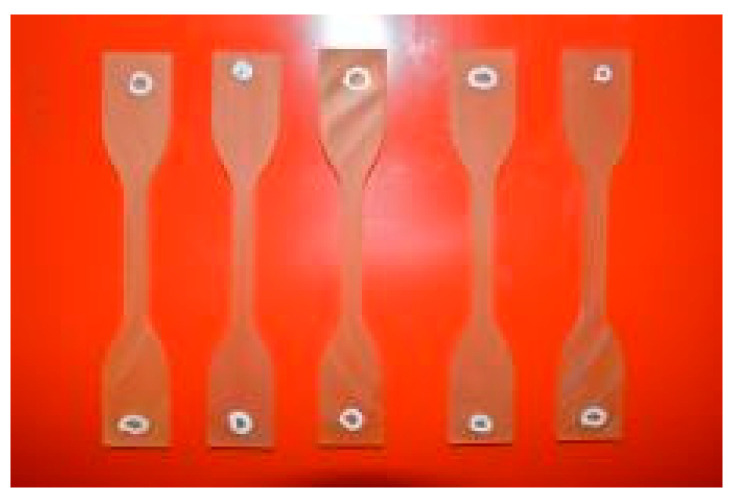
Tensile test samples of epoxy resin.

The tensile properties of the epoxy samples were tested before and after the immersion. According to ASTM D 638-2010, the testing speed was 2 mm/min. For each condition, five samples (Figure 2) were tested, and the average value was reported. The calculation of tensile strength was performed as follows:

(7) $$\sigma =\frac{F}{bh}$$

where *F* is the tensile breaking load, *b* is the width of the sample, and *h* is the thickness of the sample.

## 3. Results and Discussion

### 3.1. Water Absorption and Diffusion 

**Figure 3:**
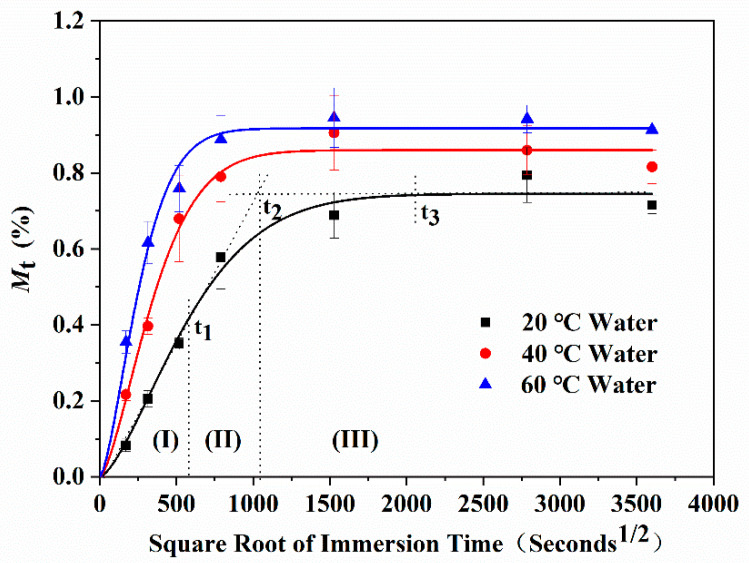
Water uptake curves of the epoxy resin immersed in distilled water at different temperatures. Solid lines represent the fitting curves using Fick’s model.

Immersed in water for a long time, samples showed obvious water absorption and weight gain, as shown in Figure 3. With the increase in immersion time, the water uptake of the epoxy resin increased linearly at first, and then gradually tended to be gentle. This process conformed to Fick’s diffusion model of the concentration gradient [23,24,25]. According to Fick’s model, the water uptake can be expressed as follows:

(8) $$M_{t}=M_{\infty }\left\{1-exp\left[-7.3{\left(\frac{Dt}{h^{2}}\right)}^{0.75}\right]\right\}$$

where *M_t_* is the water uptake at immersion time *t*, *M*_∞_ is the equilibrium water content, *D* is the diffusion coefficient, and *h* is the thickness of the specimen (2 mm in the present study).

According to the variation rate, the water uptake curves could be divided into three stages: (I) the rapid water absorption stage, the water uptake increased rapidly in a short time and was proportional to $\sqrt{t}$; (II) the increase rate of water absorption was gradually slower and the water uptake continued to increase with the extending of immersion time; (III) the saturated water absorption stage, the water uptake gradually tended to saturate with the further extending of immersion time. 

It could be also observed that when the immersed temperature increased, the starting time of the above three stages was significantly advanced. The water uptake of each stage was different, as shown in Table 1. It was considered that the absorption and diffusion of water molecules in epoxy resin were not only related to free volume, solution concentration, and other single factors, but also influenced by many factors and interactions, including concentration gradient diffusion, hydrolysis reaction, plasticization, time-temperature effect of molecular segment motion, free volume of system, defects, voids, and so on [26,27,28]. In the rapid water absorption stage, with the increase in immersed temperature, the stage time (*t*_1_) was obviously advanced, but the total water uptake increased only slightly. It was considered that the time of this stage was relatively short, and the increase in water uptake was mainly due to the hydrolysis of free water molecules in the solution and functional groups on the surface of the resin. At the same time, water molecules rapidly adsorbed on the pores and holes on the surface of the resin under the action of environmental pressure and the concentration gradient. With the increase in temperature, the physical diffusion rate of water molecules was promoted, and the water uptake of the resin increased. However, the total water uptake was mainly affected by the content of hydrolysis functional groups on the surface of the resin and the content of the pores. When the immersed temperature increased, the stage time (*t*_2_) was also advanced, and the total water uptake increased significantly. It was considered that there were double functions of “free” water and “combined” water in this stage. On the one hand, the “free” water molecules in the solution gradually entered the internal free volume space through the gaps and defects of the resin. On the other hand, the “combined” water destroyed the hydrogen bonds between the epoxy resin molecular chains or hydrolyzed with the characteristic functional groups of the resin, which could have enhanced the mobility of the small molecular segments and further formed internal micropores. With the extending of immersion time or the increase in solution temperature, the penetration of water molecules and the movement of molecular segments of the resin were significantly enhanced, and the water absorption was accelerated, which resulted in the significant increase in the total water uptake. In the saturated water absorption stage, with the increase in solution temperature, the stage time (*t*_3_) was advanced, and the water uptake was further increased than that in the previous stage and tended to be stable after *t*_3_. At this stage, with the increase in time and temperature, the movement of resin molecular segments gradually transformed to a new equilibrium state, during which new free volumes and micropores were generated and gradually occupied by water molecule. After *t*_3_, the water permeability gradually saturated, and the water uptake increased slowly to a stable level.

**Figure 4:**
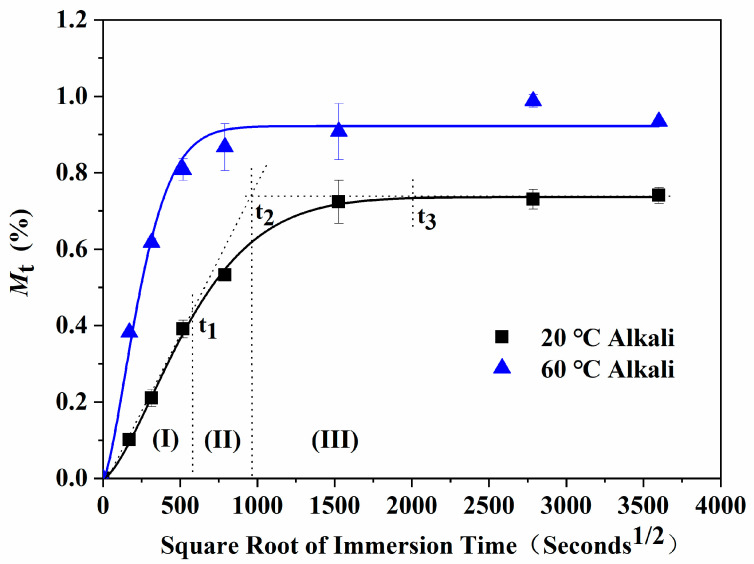
Water uptake curves of the epoxy resin specimens immersed in the alkali solution at different temperatures. Solid lines represent the fitting curves using Fick’s model.

The water absorption of epoxy resin after immersion in the alkali solution at different temperatures is shown in Figure 4. As shown, it was very similar to that of distilled water (Figure 3), which was basically consistent with previous reports. The water uptake of epoxy resin immersed in the alkali solution was in good accordance to Fick’s model [21,26].

**Figure 5:**
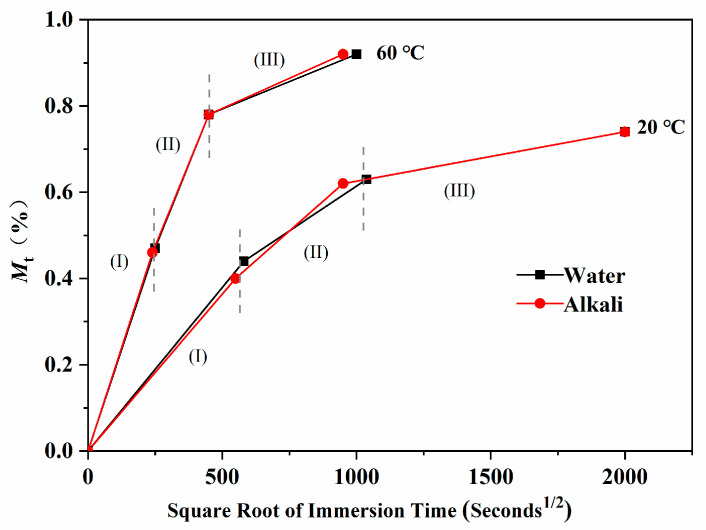
Water uptake characteristics of epoxy resin specimens immersed in distilled water or the alkali solution at 20 °C and 60 °C.

The water uptake of the epoxy resin immersed in the alkali solution at all three stages was compared with that in distilled water. The results are shown in Figure 5. For the immersion at 20 °C in the alkali solution, the stage times *t*_1_ and *t*_2_ were slightly earlier than in the water, and the water uptake of corresponding stages was relatively lower. It was considered that alkali could promote the hydrolysis reaction and accelerate the water penetration. Even the stronger hydrolysis reaction would cause the small molecules to separate from the molecular chain. This resulted in the decrease in the moisture uptake and weight gain of the resin in the alkali solution than of that in water, which led to the relatively low calculation value of the resin water uptake in the alkali solution. The situation was similar to that in the 60 °C alkali solution. According to the influence extent of alkali on the water uptake of epoxy resin, it was proposed that the proportion of hydrolysis reactions in the epoxy resin system was relatively low, which was mainly due to the effect of physical action on the absorption and diffusion of water molecules.

### 3.2. Chemical Functional Groups Analysis

**Figure 6:**
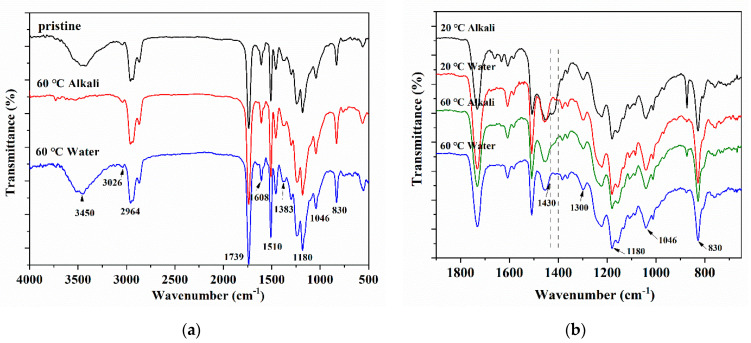
FTIR of epoxy resin specimens immersed in distiller water or the alkali solution for 150 days of (**a**) Overall infrared spectrum and (**b**) local infrared spectrum of groups below 1800 cm^−1^.

FTIR spectra at 500–4000 cm^−1^ revealed the chemical structural transition of epoxy resin before and after the immersion, which is shown in Figure 6. The characteristic groups corresponding to each peak in the infrared spectrum of epoxy resin after immersion are shown in Table 2. Figure 6a compares the immersed sample with the initial sample. As can be seen, the epoxy resin immersed in 60 °C water or alkali solution had no obvious new characteristic vibration peaks. Figure 6b shows the infrared spectrum measurements from 600–1800 cm^−1^, which showed that the resin system immersed in the alkali solution had obvious saturated alcohol OH bending vibration peaks (about 1430–1400 cm^−1^). This further proved that alkali could promote the hydrolysis reaction of the resin system.

### 3.3. Viscoelastic Behavior Analysis

**Figure 7:**
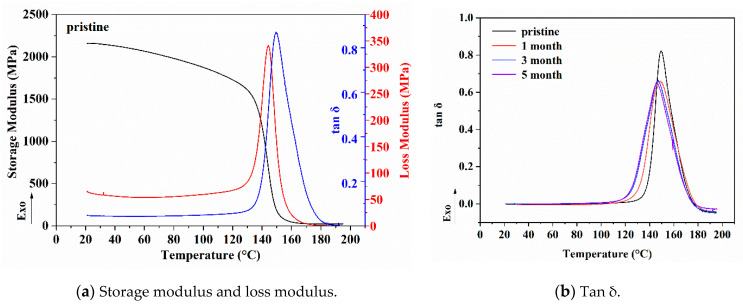
Variation of storage modulus, loss modulus of control, and tan δ of epoxy resin specimens immersed in the 60 °C alkali solution with the immersion time of (**a**) storage modulus, loss modulus of control, and (**b**) tan δ of immersed samples.

Dynamic mechanical analysis was used to investigate the effect of solution immersion on the viscoelastic behavior of the epoxy resin. The variation of storage modulus, loss modulus of control, and tan δ of epoxy resin specimens immersed in the 60 °C alkali solution with the immersion time is shown in Figure 7. As can be seen, the peak temperature of the tan δ curve of the initial specimen was about 150 °C, which represented the glass transition temperature of the control epoxy resin. After the immersion in the alkali solution at 60 °C of 1 month, the peak value of the tan δ decreased significantly, and the peak position moved to the low temperature. After the immersion of 3 months, the peak value remained basically unchanged. However, the peak position slightly decreased, and the half-maximum width of the tan δ peak slightly widened. After the immersion of 5 months, the tan δ curve had no obvious change. 

A relevant investigation proposed that the water absorption of epoxy resin was the main factor of energy loss through the plasticization effect of the molecular chain structure [28]. As seen in Figure 7, the tan δ curve of the epoxy resin immersed in the alkali solution at 60 °C could completely correspond to the variety tendency of water absorption of the epoxy resin immersed in same solution (Figure 4). It further verified the movement characteristics of the epoxy resin molecular segments during the immersion process. Epoxy resin samples immersed in the alkali solution at 60 °C for 1 month corresponded to the saturated water absorption stage. At this time, the water absorption had reached the saturation value. Before that, the epoxy resin was mainly susceptible to the hydrolysis reaction and plasticization from “combining” water. On the one hand, a few small molecular segments were hydrolyzed and broken. On the other hand, the plasticization promoted the local stretching, vibration, and displacement of random small molecular segments. This led to the appearance of new free volumes, micropores, and microcracks in the system, gradually filled with new “free” water molecules. After the sufficient time, the movement of the molecular segments of the resin basically reached a new equilibrium state, and the tan δ of the resin decreased significantly. After the immersion of 3 months, the tan δ curve of the sample changed slightly compared with that of the samples immersed for 1 month. According to Figure 4, the water absorption of the resin did not change at this time. The results showed that the molecular chain motion of the resin reached the equilibrium and stable states after the immersion in the alkali solution at 60 °C for 1 month. At this stage, the water absorption of the samples was saturated, and the tan δ changed slightly. This was mainly due to the further relaxation of the small molecular chain with the time extension. After the immersion of 5 months, the tan δ curve had no obvious change, which indicated that the tan δ of the resin was stable. The structure of the epoxy resin molecular chain after the saturated water absorption at 60 °C had reached the equilibrium state.

### 3.4. Internal Strain Monitoring

**Figure 8:**
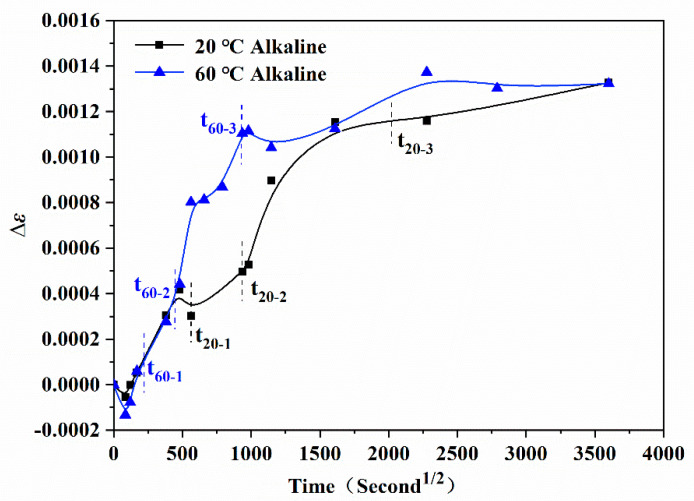
Variation of internal strain of epoxy resin specimens immersed in the alkali solution at 20 °C and 60 °C.

By monitoring the strain change in the epoxy resin embedded with fiber Bragg grating, the swelling process of the epoxy resin system and its effect on the movement of molecular segments could be directly revealed, as shown in Figure 8. As shown, the system swelled with the diffusion of water molecules into the epoxy resin. However, the crosslinking molecular chain structure of the epoxy resin would restrict the swelling of the system below the glass transition temperature, which caused the macro volume of the epoxy resin to not expand, even though the internal stress of the system increased. With the extension of immersion time, the strain of the system increased gradually at first and then tended to be stable, which was similar to the variation of water absorption with immersion time. Corresponding to the three-stage change of water uptake, the strain characteristics of the epoxy resin in each stage immersed in the alkali solution at different temperatures were further analyzed to reveal the movement tendency of molecular segments. 

Table 3 shows the strain of the epoxy resin samples immersed in the alkali solution at different temperatures. As shown, in the rapid water absorption stage the time t_1_ of the 60 °C alkali solution was shorter than that of the 20 °C solution. It was considered that the entering of water molecules into the resin system affected the movement of molecular segments and then affected the strain, which had a time effect. Because the action time of the t_1_ segment of the 60 °C alkali solution was very short, and the motion of the molecular chain segment was weak, the influence on the internal deformation of resin in this stage was negligible. The water absorption per unit time and strain in the 0–t_1_ period were higher in the 60 °C alkali solution than in the 20 °C solution (water absorption of the 60 °C alkali solution was 0.029%/h, strain was 0.0063%/h; water absorption of the 20 °C solution was 0.0048%/h, strain was 0.0036%/h). In the stages of water absorption slowing down and water absorption saturation, the water absorption of the 60 °C alkali solution was significantly higher than that of the 20 °C solution. This promoted the stronger mobility of the molecular chain segments and the larger strain of the corresponding system. After t_3_, the water absorption of the resin system tended to be saturated, and the movement of the molecular segments of the resin basically reached a new equilibrium state. At this time, the strain of the system continued to increase slowly with time, which was considered to be the effect of the relaxation of the molecular segment.

### 3.5. Free Volume Variation

The free volume change of the epoxy resin after the water absorption and diffusion was characterized by PALS. In order to reduce the influence of chemical reactions on the free volume, the epoxy resin samples were immersed in distilled water for the measurements. As shown in Table 4, the lifetime τ_3_ and the corresponding intensity *I*_3_ of the spin triplet positron beam (o-Ps) were used to characterize the size and concentration of the free volume hole, where R is the average radius of the free volume hole calculated from the lifetime τ_3_. It could be seen that compared with the initial sample, the τ_3_ value of the epoxy resin after the immersion was relatively low. With the increase in immersed temperature, the τ_3_ value further decreased and the corresponding *R* value changed in the same way. While the *I*_3_ value of the epoxy resin after the immersion was relatively high, the value of *I*_3_ increased further with the increase in immersed temperature. The results indicated that water molecules absorbed into the epoxy resin gradually occupied the free volume of the system so that the pore size (τ_3_ and *R*) decreased. With the increase in immersed temperature, the water absorption of the resin increased, and the free volume pore size further decreased. However, due to the influence of hydrolysis and plasticization, the epoxy resin would generate the new free volume holes after absorbing water, which led to the increase in the free volume concentration (*I*_3_). With the increase in immersed temperature, the mobility of molecular segments was relatively enhanced, and the corresponding free volume concentration was relatively larger. The total free volume size (*f_app_*) was obtained by combining the free volume pore size and concentration. Overall, compared with the initial sample, the free volume (*f_app_*) of the epoxy resin after immersion decreased significantly. When the immersed temperature increased, the free volume decreased further. This result showed that the water absorption into the epoxy resin after immersion at 60 °C occupied the free volume of the system more fully. 

The free volume pore size (τ_3_ and *R*) of the epoxy resin after drying was slightly lower than that of the initial sample, but the free volume concentration (*I*_3_) was significantly increased, as well as the overall free volume (*f_app_*). This indicated that the promotion of water molecules on the movement of epoxy molecular segments could effectively reduce the large-scale free volume of the system and generate more small-scale free volume. This process promoted the decrease in storage modulus of the epoxy resin system, which was consistent with DMA data (Figure 6). The results of polished samples showed that the free volume pore size (*τ*_3_ and *R*) in the resin was slightly lower than that on the surface, the free volume concentration (*I*_3_) was relatively higher, and the overall free volume (*f_app_*) was larger. 

### 3.6. Tensile Strength Variation

**Figure 9:**
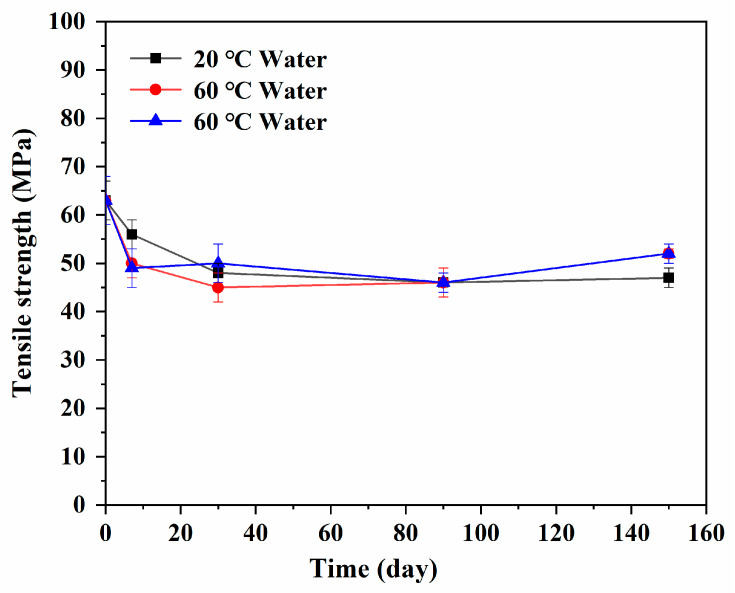
Tensile strength of epoxy resin immersed in distilled water or the alkali solution for different times.

When the epoxy resin was immersed in water or the alkali solution, the structural variation of the epoxy had a certain impact on its tensile strength. Figure 9 shows the tensile strength of the sample immersed in water solution at 20 °C. It can be seen that the tensile strength decreased rapidly with the extension of immersion time and tended to be stable after the immersion of 30 days, which was consistent with structure change owing to the absorption and diffusion of water molecules in the epoxy resin. At this stage, the water absorption increased continuously, while a small amount of hydrolysis reaction occurred in the polymer chain and some new micropores or microcracks were gradually generated. At the same time, the mobility of some molecular segments was gradually enhanced due to the plasticization. The original large-scale free space of the system gradually decreased, and the new small-scale free space began to increase, followed by the gradual movement to the epoxy resin. The tensile properties of the resin decreased obviously due to the obvious distribution of the inner free space. After the immersion of 30 days, the water absorption of the epoxy resin was determined to be saturated, and the movement of polymer segments basically reached a new equilibrium state. With the extending immersion time, the changes in water uptake and movement of molecular segments were small, and the corresponding tensile strength tended to be gentle. The tensile strength of the epoxy resin decreased by about 25% compared with the initial value after the immersion in water for 150 days. At 60 °C, the tensile strength of the epoxy resin immersed in distilled water was similar to that at 20 °C. After the immersion of 30 days, the water absorption of the epoxy resin was saturated, and it was significantly higher than that at 20 °C. The corresponding tensile strength was relatively lower than that at 20 °C. However, after the immersion of 30 days, the tensile strength of the epoxy resin increased slowly with the extension of immersion time. After the immersion of 150 days, it decreased about 17% compared with the initial sample. The tensile strength of the epoxy resin immersed in the 60 °C alkali solution for 150 days was basically the same as that of the epoxy resin immersed in the 60 °C water solution for the same time. Comparing with the increased percentage of tensile strength (∆σ) of the epoxy resin after the immersion of 150 days with that after the immersion of 90 days, ∆σ was 1.5% in the 20 °C water solution and 17.5% in both the 60 °C water and alkali solutions. It was considered that the relaxation of the epoxy resin molecular chains would further evolve to a more stable state with the extension of time. Compared with the water immersion at 20 °C, the relaxation effect of the epoxy resin molecular chains at 60 °C was more obvious. It was considered that the molecular chain structure of the resin would be further adjusted to a more stable state at a higher temperature and sufficient time. It would improve the tensile strength of the resin slightly. Although the alkali could promote the hydrolysis reaction, previous analysis found that the hydrolysis reaction was small. For the epoxy resin crosslinking by polymer chain network, it could only cause hydrolysis detachment of small molecules, and the effect on the overall structure was not obvious. The tensile strength of the epoxy resin after the immersion of 150 days was basically consistent with the water environment under the same immersion time.

## 4. Conclusions

In the present study, epoxy resin was immersed in water and an alkali solution at 20 °C, 40 °C, and 60 °C for different times. The variation of water uptake, chemical structure, thermal properties, strain, and free volume of the epoxy resin with the immersion were evaluated. The water concentration diffusion and the variations of the chemical and physical structures of the epoxy resin were investigated. The influence mechanism of structural evolution of the epoxy resin on the tensile strength was clarified. The following conclusions can be drawn:

(1) The water absorption curves of the epoxy resin immersed in water and the alkali solution have good accordance to Fick’s law. Furthermore, the water absorption curves over time presented three characteristic stages, including a rapid water absorption stage, slow growth stage, and saturated stage. Under the coupling effects of the concentration gradient diffusion, hydrolysis reaction, and molecular segment movement, the water absorption showed unique characteristics with the immersion time and temperature.
(2) After the immersion in each solution for one month, the water absorption of the resin was determined to be saturated, and the viscoelasticity of the system decreased significantly. When the immersion time was further extended, the water absorption did not change while the relaxation of the molecular segments of the resin was further developed. The original large-scale free space of the resin gradually decreased, and the new small-scale free space began to increase and gradually distributed to the interior of the epoxy resin.
(3) The tensile strength of the resin decreased with the increase in water absorption until the lowest value after the immersion of one month. Compared with the initial sample, the decreased percentages of tensile strength in 20 °C and 60 °C water or the 60 °C alkali solution for one month were 24%, 28%, and 22%, respectively. After that, the tensile strength recovered with the further extension of immersion time, owing to the molecular chain structure of the resin that would be further adjusted to a more stable state at higher temperature and sufficient time. In addition, the effect of the alkali solution and water solution on the tensile strength of the epoxy resin was basically the same.

## Figures and Tables

**Table 1:** Water absorption characteristics of epoxy resin immersed in distilled water at different temperatures.

Immersion Temperature (°C)	Stage I		Stage II		Stage III	
	*t*_1_ (s^1/2^)	$M_{t}$ (%)	*t*_2_ (s^1/2^)	$M_{t}$ (%)	*t*_3_ (s^1/2^)	$M_{t}$ (%)
20	580	0.44	1038	0.63	2000	0.74
40	300	0.45	625	0.72	1250	0.86
60	250	0.47	450	0.78	1000	0.92

**Table 2:** Characteristic groups corresponding to each peak of the infrared spectrum of epoxy resin after immersion [29].

Peak (cm^−1^)	Characteristic Functional Groups	Peak (cm^−1^)	Characteristic Functional Groups
3450	–OH stretching	1430–1400	OH bending of saturated alcohol
3026	Hydrocarbon CH stretching	1300	C–O cable
2964	CH stretching (antisymmetric)	1200–1100	CO cable of alcohol
1739	C=O stretching	1046	Stretching of phenyl ether bond
1608	Benzene skeleton	830	Bending between adjacent hydrogens on benzene ring
1510	Benzene skeleton		

**Table 3:** Strain of epoxy resin samples immersed in the alkali solution at different temperatures.

Immersion Solution	(I) Stage (0~T_1_)		(II) Stage (T_1_~T_2_)		(III) Stage (T_2_~T_3_)		After Stage	
	$M_{t}(\%)$	Δε(%)	$M_{t}(\%)$	Δε(%)	$M_{t}(\%)$	Δε(%)	$M_{t}(\%)$	Δε(%)
20 °C alkali	0.40	0.03	0.22	0.02	0.12	0.06	0.01	0.02
60 °C alkali	0.46	0.01	0.32	0.03	0.14	0.07	0.01	0.02

**Table 4:** Free volume change of epoxy resin after the water absorption saturation at different temperatures.

Parameter	Initial Sample	W-20	W-60	W-20 (Drying)	W-40 (Polished)
τ_3_ (ns)	1.8807	1.8523	1.8279	1.8692	1.8411
*R* (nm)	0.274	0.271	0.269	0.273	0.270
*I*_3_ (%)	26.338	26.4492	26.6104	26.7063	27.0773
*f_app_* (nm)	2.269	2.205	2.169	2.276	2.232

Note: W-20 in the table represents the distilled water environment at 20 °C, and the rest was the same.

## Data Availability

The data presented in this study are not available.
